# Atrial fibrillation driver identification through regional mutual information networks: a modeling perspective

**DOI:** 10.1007/s10840-021-01101-z

**Published:** 2022-01-04

**Authors:** Qun Sha, Luizetta Elliott, Xiangming Zhang, Tzachi Levy, Tushar Sharma, Ahmed Abdelaal

**Affiliations:** 1 Medical Affairs, Biosense Webster, Irvine, CA 92618 USA; 2 Research and Development, Biosense Webster, Irvine, CA 92618 USA

**Keywords:** Atrial fibrillation, Electrical drivers, Rotational activation, Cardiac mapping, Mutual information, Local efficiency

## Abstract

**Purpose:**

Effective identification of electrical drivers within remodeled tissue is a key for improving ablation treatment for atrial fibrillation. We have developed a mutual information, graph-based approach to identify and propose fault tolerance metric of local efficiency as a distinguishing feature of rotational activation and remodeled atrial tissue.

**Methods:**

Voltage data were extracted from atrial tissue simulations (2D Karma, 3D physiological, and the Multiscale Cardiac Simulation Framework (MSCSF)) using multi-spline open and parallel regional mapping catheter geometries. Graphs were generated based on varied mutual information thresholds between electrode pairs and the local efficiency for each graph was calculated.

**Results:**

High-resolution mapping catheter geometries can distinguish between rotational and irregular activation patterns using the derivative of local efficiency as a function of increasing mutual information threshold. The derivative is decreased for rotational activation patterns comparing to irregular activations in both a simplified 2D model (0.0017 ± 1 × 10^−4^ vs. 0.0032 ± 1 × 10^−4^, *p* < 0.01) and a more realistic 3D model (0.00092 ± 5 × 10^−5^ vs. 0.0014 ± 4 × 10^−5^, *p* < 0.01). Average local efficiency derivative can also distinguish between degrees of remodeling. Simulations using the MSCSF model, with 10 vs. 90% remodeling, display distinct derivatives in the grid design parallel spline catheter configuration (0.0015 ± 5 × 10^−5^ vs. 0.0019 ± 6 × 10^−5^, *p* < 0.01) and the flower shaped open spline configuration (0.0011 ± 5 × 10^−5^ vs. 0.0016 ± 4 × 10^−5^, *p* < 0.01).

**Conclusion:**

A decreased derivative of local efficiency characterizes rotational activation and varies with atrial remodeling. This suggests a distinct communication pattern in cardiac rotational activation detectable via high-resolution regional mapping and could enable identification of electrical drivers for targeted ablation.

**Supplementary Information:**

The online version contains supplementary material available at 10.1007/s10840-021-01101-z.

## Introduction

Atrial fibrillation (AF) is the most common arrythmia, projected to affect 6–12 million people in the USA by 2050 and 17.9 million people in Europe by 2060 [1]. Catheter ablation is a treatment option for symptomatic AF which aims to isolate or eliminate dominant electrophysiological substrates. A variety of substrate mapping and ablation strategies have been proposed to achieve this goal, including the identification of electrical drivers [2, 3]. The term “ AF driver” or “Electrical rotational activation” or “Rotor” applies to organizing source of vortex-like functional reentry, as a curved wave that rotates around an unexcited core and forms a spiral shape [4]. AF driver identification using the current electroanatomic mapping system has been a challenge: arrythmia cycle lengths and tissue heterogeneity can obscure re-entrant circuits within complex electrograms. The mapping catheters in the present iterations have demonstrated only limited clinical utilities for guiding ablations, due to their poor spatial temporal resolution and restricted software modules. This has resulted in repeat ablations as well as variability in efficacy among patient groups [5, 6]. It is therefore critical to develop novel strategies for better identifying target electrical drivers.

Insight into AF mechanism classification can be gained by approaching cardiac arrhythmias from a network theory perspective. Networks, collections of nodes connected by edges, have been widely used to represent relationships in systems such as social networks, metabolism, and the brain. Networks find a natural application in mapping cardiac arrhythmias: tissue areas surrounding an electrode can be represented as nodes while various relationships between neighboring electrical signals can be used to define edges. This approach has been used to demonstrate changes in global tissue connectivity upon ablation [7]. Networks can also be used to detect AF driver, by establishing links between electrodes based on sequential local activation time and applied a standard search algorithm for rapid cycle detection [8].

Mutual information is a measure of dependence between two signals: the amount of information obtained about one signal based on the observation of another. This measure has been applied to profiling communication between cardiomyocytes during arrhythmias, demonstrating spatially heterogeneous patters during sinus rhythm, focal activation, and spiral reentry [9]. Spiral wave dynamics in particular can effectively be quantified through mutual information and correlated to micro-scale behavior of cardiac system components [10, 11].

**Fig. 1:**
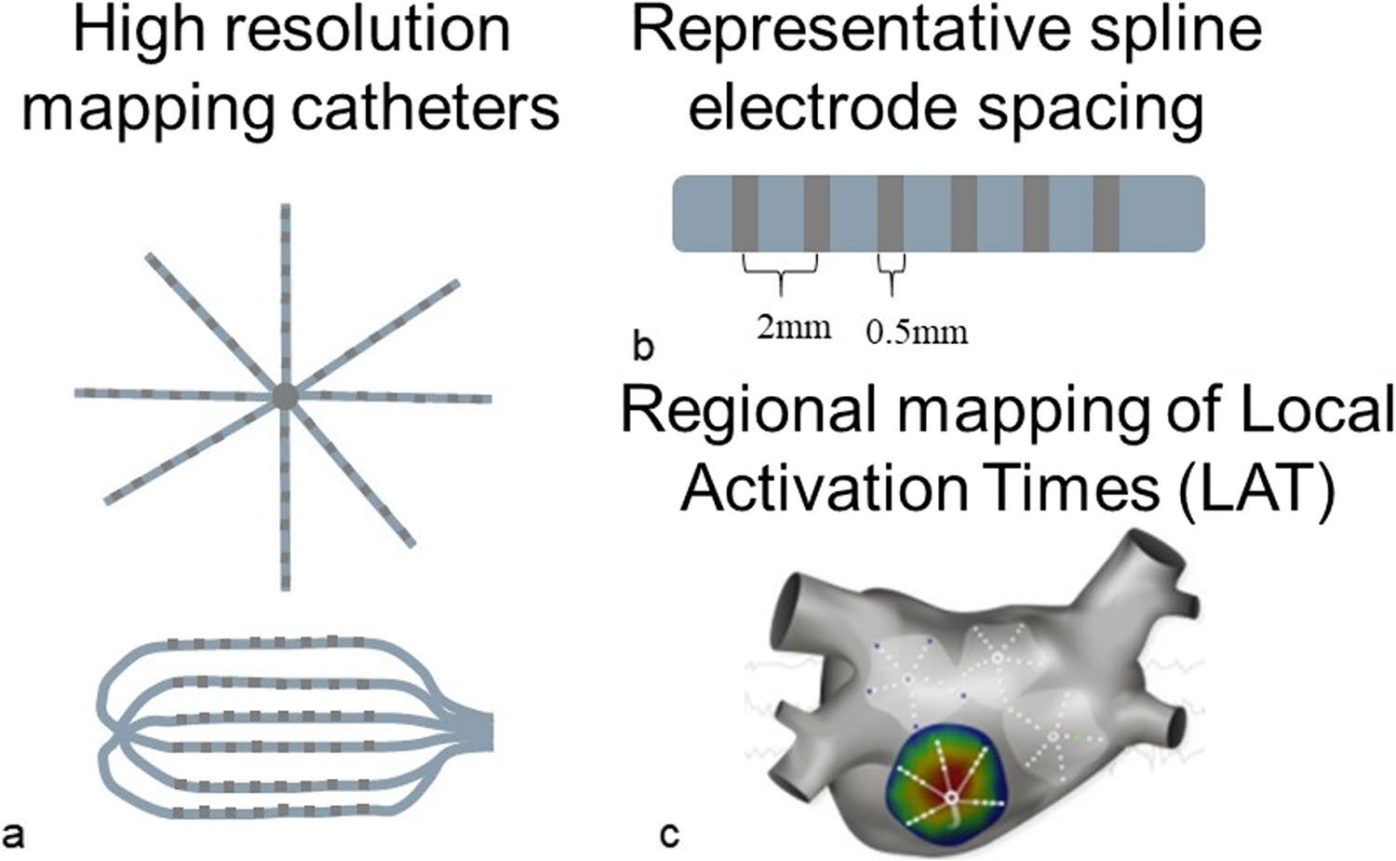
Clinical regional high-resolution local activation time mapping. **a** A schematic of high-resolution mapping catheters with flower-shaped open spline and grid-type parallel spline configurations. **b** A schematic of a catheter spline representing mapping electrodes and the spacing between them. **c** A schematic representing regional local activation time maps obtained throughout the atrium with a high-resolution flower-shaped open spline catheter. The colored region represents an individual map

The high-resolution multi-spline mapping catheter technology provides the opportunity to apply network and mutual information approaches to AF driver detection. Periodic stationary rotors can process in 2–3 cm^2^ areas, making electrogram points obtained from regions of comparable area, rather than the whole atrium, particularly attractive for the real time 3D electroanatomic mapping [12]. Sequential mapping with the catheters that cover 7.1 cm^2^, combined with automated activation detection software, can identify temporally stable activations associated with AF termination (Fig. 1) [13, 14]. These mapping catheters also enable the activation identification within highly scattered regions that otherwise appear electrically silent [15]. The advanced mapping therefore provides rich regional information for further network and information theory-based analysis.

In this report, mutual information graphs are applied to gain insight into the regional electrical driver in AF. Using 2D and 3D atrium computational models, we identify a metric that distinguishes between irregular and rotational activity at this scale—local efficiency. We then analyze an atrial tissue model that incorporates varying degrees of electrophysiological remodeling, demonstrating the utility of local efficiency in identifying drivers based specifically within remodeled substrates. Local efficiency, as a graph theory metric of fault tolerance, demonstrates the interference of tissue remodeling in regional communication and presents an avenue for identification of drivers located specifically within atrial electrical remodeling.

## Materials and methods

**Fig. 2:**
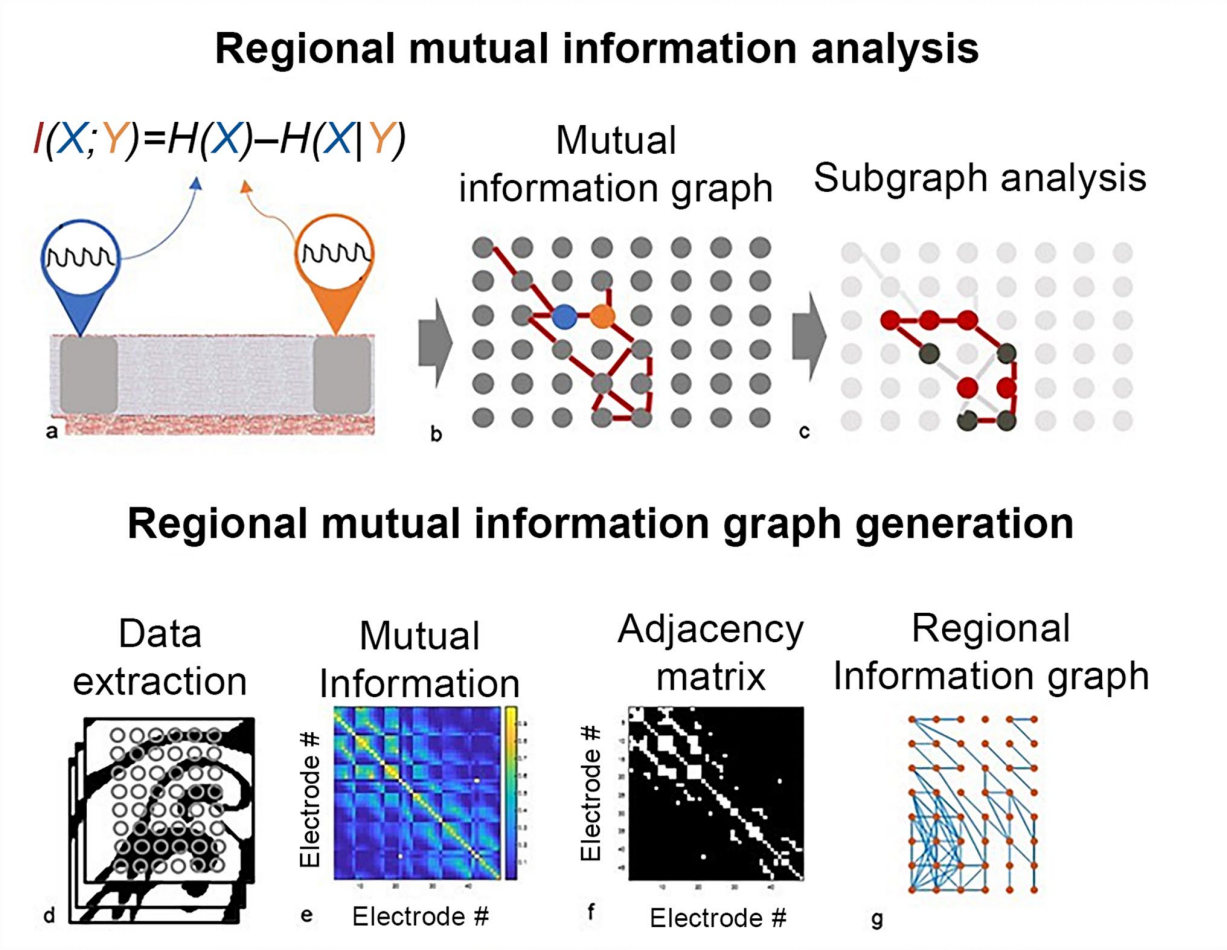
Regional mutual information graph approach. **A**) A schematic representing two mapping electrodes positioned on cardiac tissue. The action potentials at the electrode locations are used to estimate the mutual information between the recording sites. **B**) A graph with each node representing a recording electrode, with electrodes from panel A highlighted. The connections between nodes (edges) represent mutual information values above a set threshold. **C**) A schematic illustrating subgraph analysis. A set of nodes connected to a single central node thereby forming a subgraph is highlighted in dark grey. Edges highlighted in red represent the shortest paths between subgraph nodes that do not connect to the central node. The average of these path lengths over multiple subgraphs represents local efficiency. **D**) A series of electrode positions shown in grey overlayed over a series of simulated voltage maps demonstrate data extraction using a particular electrode geometry. **E**) A mutual information matrix demonstrating the mutual information between each pair of electrodes. Increased mutual information is shown in yellow. **F**) A binary adjacency matrix calculated by setting a threshold for mutual information between electrode pairs. **G**) A graph with each node (orange) representing an electrode within a mapping catheter and each edge (blue) representing sufficient mutual information between electrode pairs based on the adjacency matrix

A mutual information-based analysis was developed for regional voltage maps at the scale accessible with high-resolution multi-spline catheters of varied geometries. Simulated electrical driver data for analysis was first generated. Mutual information was then calculated based on action potential (AP) voltage signals obtained from any electrode pair within the catheter (Fig. 2a) to generate a graph where connections between electrodes if a particular mutual information threshold are reached (Fig. 2b). The subsequent graph analysis was focused on local efficiency, which is a metric calculated based on the average path length between electrodes of a given subgraph (Fig. 2c). The details of this approach are described below and summarized in a schematic in Figure S1.

### Simulations

Multiple cardiac spiral wave simulations (2D and 3D) were adopted to verify the independence of observed graph characteristics from a particular model. In particular, the simplified 2D Karma model was selected to suggest if the analysis identified features of repeat spiral activation or of more complex physiological patterns originating from variable AP morphology. A model that represents re-modeling through Ca^2+^ dynamics was also adopted to represent a feature characteristic of cardiac tissue in persistent AF.

*2D simulations* were based on the Karma model, which is a widely used simplification of more complex ionic models of the cardiac membrane. This model incorporates fast inward, slow outward, and slow inward currents that correspond to physiological Na^+^, Ca^2+^, and K^+^ current [16]. The model can reproduce the AP duration and conduction velocity but not morphology generated by more complex models. This simplification is suitable for studying spiral wave behavior, for which the model was initial designed, due to the key role of restitution properties in determining wave behavior [17].

The simulations were performed using the virtual heart platform WebGL implementation developed for rapid cardiac simulations [18]. The 512×512 cell 2D Karma model was utilized in the spiral wave regime (nb= 1.0, m=7.0, eps= 0.4, simulation time= 10s) to extract 1500 frames of spiral activity. The set parameters determine the AP duration of a single pulse, the sensitivity of the wavefront during propagation (tendency to disperse), and the relationship between the time scale of the upstroke to the maximum AP duration, respectively. While these parameters can be adjusted to match AP duration and conduction velocity of clinical data, they cannot be independently adjusted to reflect properties such as cycle length. Spontaneous breakup of the spiral wave produces irregular activity, of which 1500 frames were also extracted. The images were converted to binary by ImageJ to enable efficient mutual information estimation during further analysis. The produced videos are available in the supplementary information (Video S1–2).

*3D analysis* was based on previous 10s 3D simulations by Rios-Munoz et al. [19]. The realistic atrial model implements heterogeneity in fiber orientation as well as anisotropy in conduction velocity and ionic currents [20]. The simulations incorporate the AF-remodeled version of the cellular model [19, 21]. Briefly, this model aims to accurately represent the repolarization process, incorporating improved formulations for the transient outward K^+^ current ultrarapid rectifier K^+^ current. These simulations considered two activity scenarios: rotor activity and irregular wavefront collisions at a sampling frequency of 1kHz. The simulation outputs were AP voltages.

The portion of the simulation output video containing a stabilized rotor core, as well as the corresponding region of irregular wavefront collision simulation, were isolated for further 2D analysis of surface AP voltages. The 1500 frames obtained from each simulation were converted to binary images via ImageJ by thresholding at −50mV to enable efficient mutual information calculations. The produced surface activation videos are available in the supplementary information (Video S3–4).

*Remodeling simulations* were performed using the Multiscale Cardiac Simulation Framework (MSCSF), a C/C++ implementation for cardiac electrophysiology simulations [22]. This modeling framework aims to represent the bi-directional coupling between sub-cellular and tissue level processes. The particular modeling parameters chosen for this study trigger rotor and included an implementation of spontaneous sub-cellular Ca^2+^ release events associated with remodeling. Specifically, first, a 1D model was paced to a stable state, using the RSERCA_NCX cell model with a conduction velocity D1 of 0.25 mm/ms, tissue modulation (Dscale) of 0.75, and cell size of 0.3 mm at a basic cycle length of 300ms for 100 beats. Following pacing, this cell model is designed to spontaneously mimic general disease remodeling features observed in AF: upregulation of the sarcoplasmic reticulum Ca^+2^ pump and downregulation of the Na^+^-Ca^2+^ exchanger. These features promote spontaneous Ca^2+^ release and result in pro-arhythmic behavior. The corresponding 2D re-entry tissue model composed of 250×250 such cells was then paced for 2 beats before applying an S1 beat and an S2 of 155. The simulation time of the final segment was set 460ms. The remodeling conditions were modulated on a linear scale, with the degree of remodeling modification applied to the cell model varied from 10 to 90% remodeling. Increasing degrees of remodeling represent downregulation of the Na^+^-Ca^2+^ exchanger which lowers the Ca^2+^ efflux, resulting in spontaneous Ca^2+^ to propagate to neighboring cells and sustained arrhythmia. This is implemented through linear scaling of model parameters, including ion flux rate ratios, maximum intracellular uptake, and the maximum Na^+^-Ca^2+^ exchanged current [22]. The obtained voltage files were imported into ParaView software and converted to binary using ImageJ for mutual information estimation.

### Regional network generation

To extract data for analysis, binary voltage signals were obtained at locations corresponding to particular electrode positions from simulated data sets. As demonstrated in a representative time slice of the analyzed 3D simulated tissue with rotational activation in Fig. 2d, close electrode positioning captured the simulated activations. The obtained binary signals were processed to calculate mutual information at each electrode pair, as demonstrated in the corresponding representative mutual information matrix in Fig. 2e. As expected, maximum mutual information is observed on the principal diagonal, which corresponds to the comparison of an electrode signal to itself. Several surrounding clusters of increased mutual information are also observed. This matrix is converted to a binary adjacency matrix by selecting a particular portion of connections with the highest mutual information, as demonstrated in Fig. 2f for the top 10% most informative connections. This thresholding, although chosen arbitrarily, unambiguously highlights clusters of high mutual information. This matrix is directly converted to a graph for further analysis (Fig. 2g).

This analysis was performed using MatLab (R2019a, MathWorks) using two investigational catheter geometries representing (a) flower-shaped radial spline placement and (b) grid-type parallel spline placement. Unless otherwise noted, electrodes in the radial spline geometry were positioned on eight 2 cm splines, each separated by 45 degrees. The 48 electrodes were 500 µm in length and spaced 2 mm apart. Electrodes in the parallel (grid) geometry were positioned on six splines spaced 2.14 mm apart. The 48 electrodes were 500 µm in length and spaced 2 mm apart. For each time point in the obtained volage map, the catheter was positioned within the analyzed tissue and voltage values from four simulation cells neighboring the electrode were averaged and rounded. This produced a binary signal sequence corresponding to each electrode. Mutual information between each bipoles was estimated using the mutual information function from the Toolbox for C and MatLab  [23]. Specifically, the estimated mutual information is provided by Eq. 1 below:

1 $$I\left(X;Y\right)\approx \frac{1}{N}\sum\nolimits_{i=1}^{N}log\frac{\widehat{p} \left({x}^{i}{y}^{i}\right)}{\widehat{p} \left({x}^{i}\right)\widehat{p} \left({y}^{i}\right)}$$

where *N* is the number of voltage signals observed at a given location for the duration of the simulation and distributions $\widehat{p}\left({x}^{i}{y}^{i}\right)$,$\widehat{p}\left({x}^{i}\right)$, and $\widehat{p}\left({y}^{i}\right)$ are obtained from fixed-width bin histogram estimators using voltage based binary sequences at a given electrode pair. The obtained mutual information matrix was converted to a binary adjacency matrix by setting various information thresholds, which represented increasing percentages of potential edges being extracted for analysis. A graph was generated for each corresponding adjacency matrix. For each catheter geometry/simulation approach combination, this analysis was performed for 6 catheter positions within the simulated tissue.

### Graph analysis

Generated graphs were analyzed using Matlab graph analysis toolbox with a focus on the local efficiency parameter [24]. Local efficiency at a given electrode is given by Eq. 2 below:

2 $$E_{local}=\frac1{N_{Gi}(N_{Gi}-1)}{\textstyle\sum_{i\neq j\in Gi}}\frac1{L_{i,j}}$$

where *G*_*i*_ is a subgraph, *N*_*Gi*_ is the number of nodes in the subgraph, and *L*_*i,j*_ is the path length between a pair of nodes. The value was averaged over all electrodes to determine the mean local efficiency of the graph.

Local efficiency for graphs corresponding to each catheter position was calculated as a function of the mutual information threshold used during graph generation. This function captured the variation in local efficiency without selecting a particular arbitrary information threshold. The derivative of the function, calculated based on the third-degree polynomial fit, was used to illustrate the relationship between changes in local efficiency and mutual information.

### Initial clinical data analysis

A CARTOFINDER system (Biosense Webster, Irvine, CA) identified rotational activation 4D LAT map (Video S5) was kindly provided by Andrea Sarkozy, MD, at Universitair Ziekenhuis Antwerpen, Belgium. This system uses the CARTO 3 electroanatomical mapping platform and creates wavefront propagation maps from unipolar signals acquired by a multiple spine mapping catheter. During a electrophysiology mapping feasibility study for a persistent atrial fibrillaiton patient (subject 841–004), a rotational activation in left atrium anterior wall was captured, when utilizing the novel flower-shaped high-density mapping catheter containing 48 platinum-iridium mapping electrodes distributed across eight spines [25].

The portion of the video corresponding to the stable rotors core outside of ablated regions was isolated for the initial proof-of-concept analysis. The 125 frames corresponding to the rotational activation were converted to binary in ImageJ by thresholding at − 153 ms 4D-LAT (local activation time) based on physician parameter selection during video recording. The corresponding original video and isolated binary segment are available in the supplementary information (videos S5–6).

From the binary.png map images generated, a value of “1” or “0” is captured by digital electrodes superimposed over the clinical data. Each electrode thus has a sequence of binary values over the video runtime. For each pair of electrodes *x* and *y*, p̂(x), p̂(y), and p̂(xy) are used to generate a pairwise mutual information matrix; The pairwise mutual information matrix can then be thresholded at various values of mutual information to generate the adjacency matrix, which is the basis of a regional information graph where edges represent sufficient mutual information between electrodes. Local efficiency was calculated at each electrode of the catheter and averaged to generate local efficiency.

## Results

### AF driver identification

**Fig. 3:**
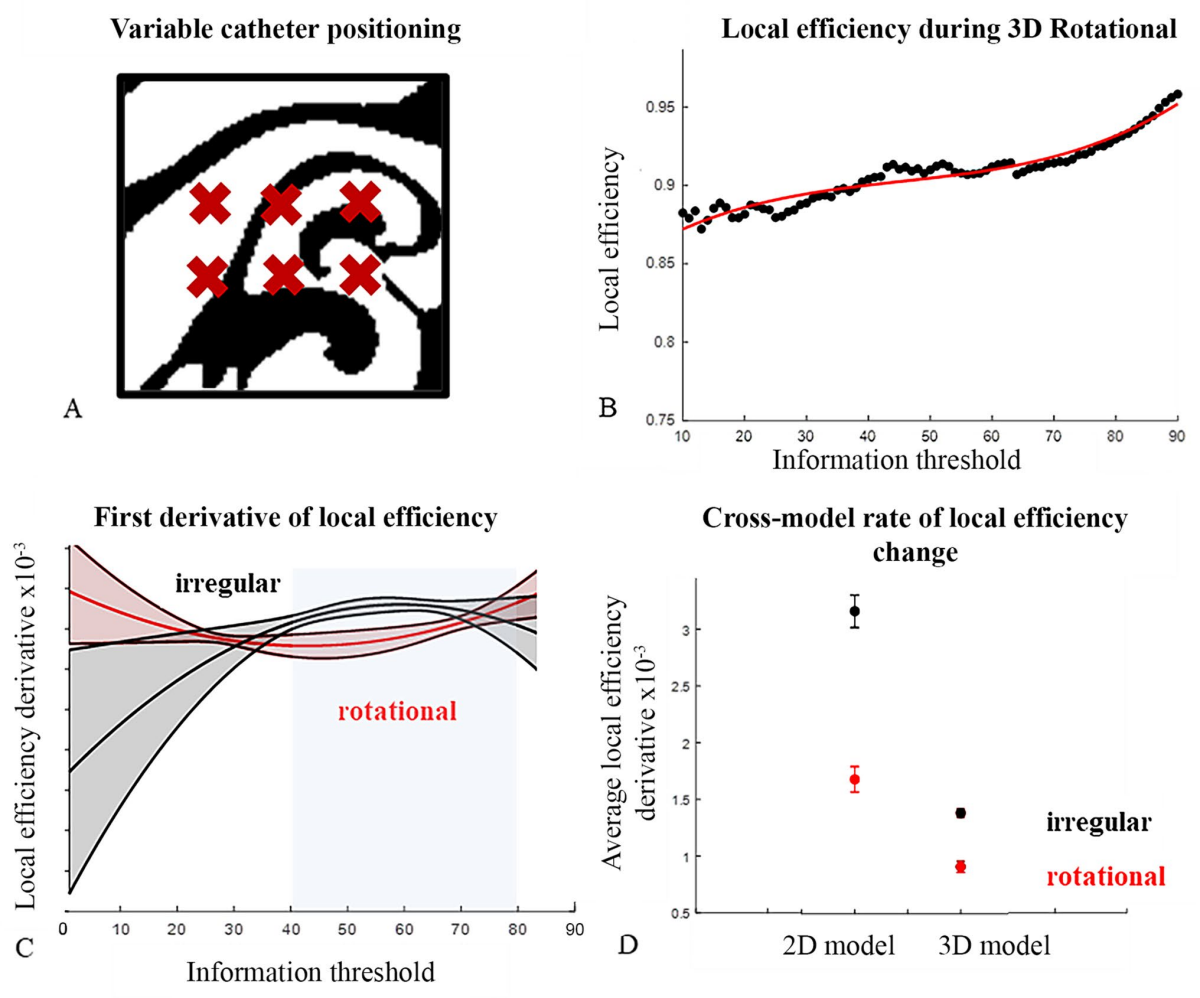
Local efficiency signature of rotational activation. **a** Schematic representation of variable catheter positioning. Red crosses represent the center of the electrode array within a single time point of simulated rotational activation in the 3D model. **b** Local efficiency as a function of information threshold plotted in black for one of six grid type catheter positions within simulated tissue (3D model rotational activation). Corresponding third degree polynomial fit is shown in red. **c** First derivative of local efficiency for 3D tissue simulations with irregular (black) and rotational (red) activation patterns. The shaded region represents the 95% confidence interval based on varied grid type catheter position within the simulated tissue (*n* = 6). The region between the 40 and 80% information threshold used in further analysis is highlighted in blue. **d** Comparison of the average first derivative in the 40–80% information threshold range for irregular (black) and rotational (red) activation patterns in 2D and 3D simulations. Error bars represent the 95% confidence interval

The method was first applied to the identification of drivers in simulated tissue, focusing on the local efficiency metric calculated as a function of the mutual information threshold used to define the adjacency matrix. We analyzed data extracted by a grid-type mapping catheter geometry from tissue modeled using the simple 2D Karma model and the more physiologically accurate 3D atrial model. In each case, six distinct catheter positions within simulated data sets were analyzed, with geometric centers positioned as illustrated in Fig. 3a for the 3D rotors case. The typical calculated local efficiency as functions of mutual information threshold for this simulation is displayed in Fig. 3b, together with third degree polynomial fit. Results for the corresponding irregular and rotational 3D and 2D simulation are available in Figure S2. Derivative calculations based on the third-degree polynomial fit (Fig. 3c) indicate an overlap in the 95% confidence interval at information threshold values below 40% and above 70% and distinct derivative values in the intermediate range for the 3D simulation results. Corresponding data for the 2D simulations is available in Figure S3, and indicate a lack of confidence level overlap in the 50–80% region. Comparison of average derivatives in the 40–80% information threshold range (Fig. 3d) indicate a significant (*p* < 0.01 using a two-sample *t*-test) decrease in the rate of rate of local efficiency change when driver is introduced: 0.0032 ± 1 × 10^−4^ vs. 0.0017 ± 1 × 10^−4^ for the 2D model and 0.0014 ± 4 × 10^−5^ vs. 0.00092 ± 5 × 10^−5^ for the 3D model. Lack of consideration of more extreme information thresholds is justified by the presence of sparse of uniformly connected matrices under these conditions.

### Electrophysiological remodeling identification

**Fig. 4:**
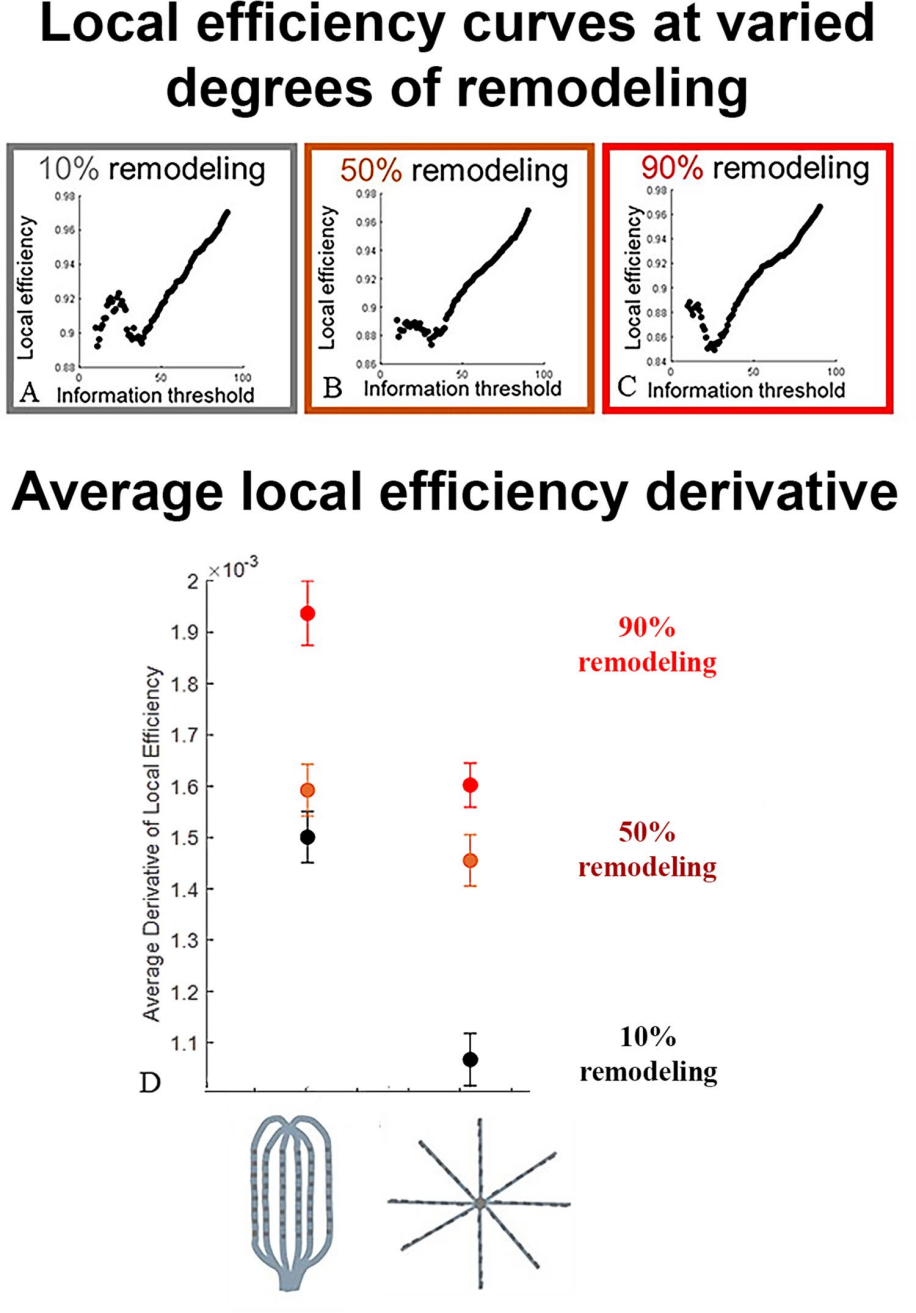
Local efficiency for remodeling classification. **A**) Representative local efficiency calculated for graphs with increasing information thresholds based on simulations that incorporate 10% remolding and a grid type catheter with 2 mm interelectrode spacing. **B**) Representative local efficiency calculated for graphs with increasing information thresholds based on simulations that incorporate 50% remolding and a grid-type catheter with 2 mm interelectrode spacing. **C**) Representative local efficiency calculated for graphs with increasing information thresholds based on simulations that incorporate 90% remolding and a grid catheter with 2 mm interelectrode spacing. **D**) The rate of change in local efficiency for 40–80% information thresholds for grid-type catheters and flower-shaped open spline-type catheters. Derivatives are averaged for 90% (red), 50% (orange), and 10% (black) remodeling simulations based on 6 catheter positions within the simulated tissue. Error bars represent the 95% confidence interval

The method was then applied to the driver generated in simulated tissue with varying degrees of remodeling. Local efficiency was calculated as a function of the information threshold for data extracted via grid type catheter geometry from simulated tissue with remodeling degrees of 10% (Fig. 4a), 50% (Fig. 4b), and 90% (Fig. 4c). Fitted local efficiency functions for varied catheter geometries and degrees of remodeling are available in Figure S4. In all cases, instability was observed below the 40% information threshold. When the rate of local efficiency change was quantified at varied catheter positions within the simulated tissues as discussed above, the average derivatives in the 40–80% information threshold ranges were compared for both grid-type parallel spline and flower-shaped open radial spline catheter geometry configurations. Increased derivatives were observed in 90% remodeled tissue as compared to 10% remodeled tissue: 0.0019 ± 6 × 10^−5^ vs. 0.0015 ± 5 × 10^−5^ for the parallel spline configuration and 0.0016 ± 4 × 10^−5^ vs. 0.0011 ± 5 × 10^−5^ for the flower-shaped open spline configuration. This indicates a significant (*p* < 0.01 using a two-sample *t*-test) difference between the local efficiency derivatives due to remodeling for both catheter geometries. An intermediate degree of remodeling of 50% resulted in intermediate derivative values: 0.0016 ± 5 × 10^−5^ and 0.0015 ± 5 × 10^−5^ for the parallel and open spline configurations, respectively.

**Fig. 5:**
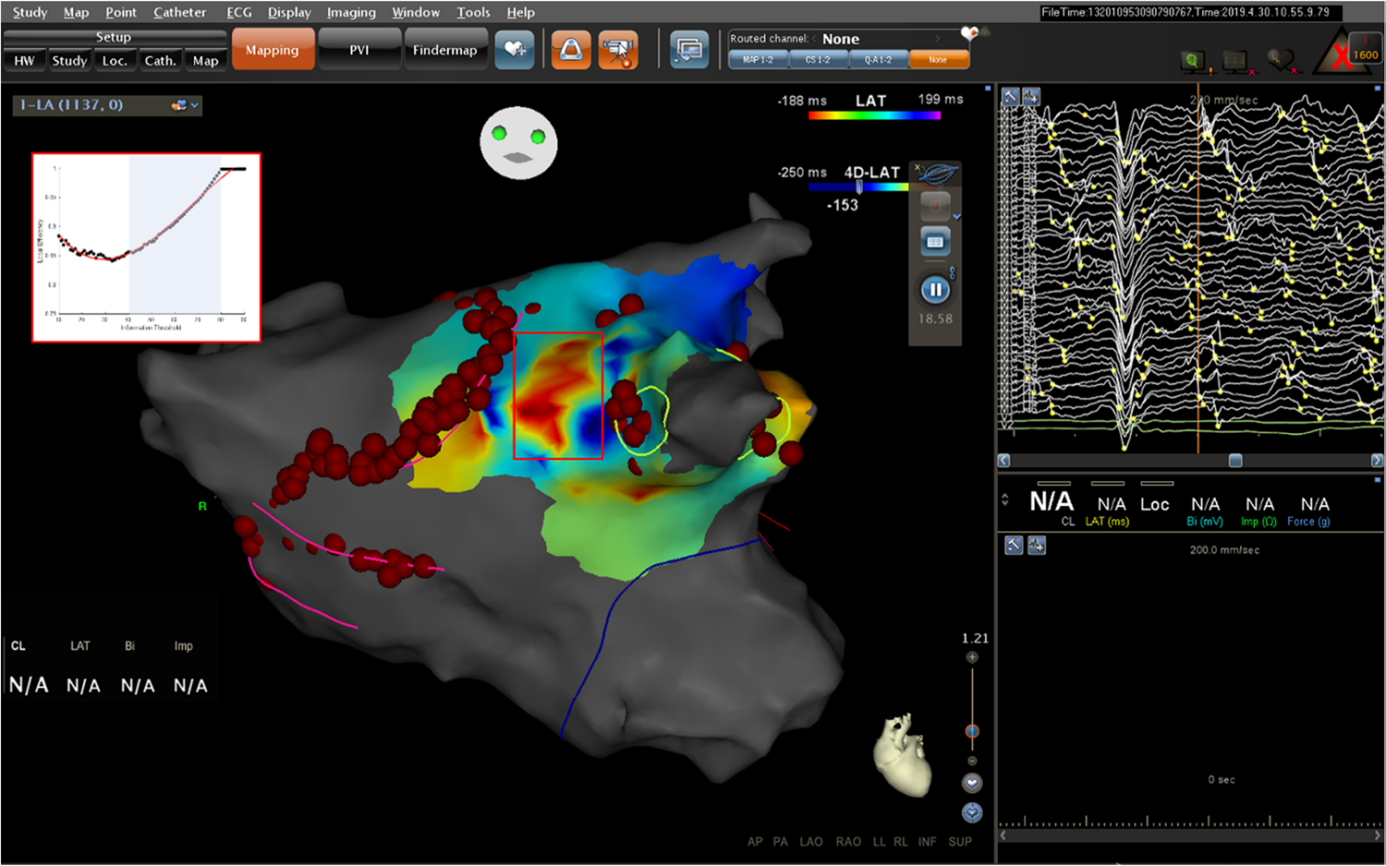
Demonstration of a local efficiency curve simulating from a clinical AF map (Subject 841–004, Sarkozy et al. 2020 [25]), which could be integrated into CARTOFINDER rotational activity analysis interface in the Carto 3 mapping system. The red rectangle within the LAT map highlights the anterior wall region of interest analyzed. The putative insert displays local efficiency values and the corresponding polynomial fit as a function of information threshold

As an initial proof of concept, local efficiency was calculated as a function of information threshold based on a clinical rotational activation video from a persistent AF mapping case using CARTOFINDER algorithm and a single overlay of flower-shaped radial spline catheter (Fig. 5). The average derivative in the 40–80% information threshold region of interest was 0.0032. Constant local information of 1 observed above the 80% information threshold corresponds to a uniformly connected matrix at that threshold.

## Discussion

### Local efficiency in atrial fibrillation

Local efficiency has previously been estimated in macaque, cat, and human brains, but has not yet been applied to AF disease states [26–28]. It signifies fault tolerance, indicating how well each subgraph exchanges information when the index electrode is eliminated. Local efficiency therefore represents the capacity of a network for parallel, or redundant, information transfer. In Fig. 3d, simulated cardiac tissue that contains a rotational pattern displays a decrease in the rate of change in this metric as a function of information threshold, irrespective of the complexity of the simulation system. It indicates that the addition of less informative connections to networks representing rotor does not rapidly increase fault tolerance. These connections, unlike those observed in irregular systems that contribute to an increase in local efficiency, represent a new characteristic of AF drivers.

### Remodeling mechanisms

AF results in progressive electrophysiological and structural changes in the atria, termed “remodeling,” which produce sustained AF that is challenging to terminate [29]. In this electrophysiological model, we demonstrate that fault tolerance changes more rapidly with the inclusion of less informative connections in highly remodeled tissues (Fig. 4). It indicates that redundant information transfer capacity in remodeled networks could depend on weakly informative connections. This suggests that the increased Ca^2+^ diffusion used to represent remodeling in this study impede parallel information transfer [22]. The variable fault tolerance may interact with other remodeling mechanism, such as structural remodeling on scar formation. Scarring plays a key role in AF maintenance: in patients undergoing pulmonary vein isolation, left atrium scarring is a strong predictor of procedure failure and increased recurrence (57%) compared to non-scared patients (19%) [6, 30, 31]. One promising ablation strategy is based on modifying low-voltage regions on non-uniform anisotropic conduction, resulting in improved outcomes after repeat procedures [32]. It is possible that variable fault tolerance observed in the Ca^2+^ diffusion-based remodeling approach of this study may decrease the ability of cardiac tissue to transmit signals around low-voltage scar areas, thereby contributing to AF maintenance. Therefore, further study of fault tolerance in models that incorporate various remodeling mechanisms is warranted.

### Implications for substrate mapping and ablation strategy

To understand AF mechanisms, different methodologies [33–36] have been developed to map electrical activity, namely body surface mapping, electrocardiographic imaging, multielectrode plaque epicardial electric mapping, multiple-spline, grid type [37], and basket catheter endocardial mapping. The innovative algorithms including spectrum analysis, complex fractionated atrial electrograms (CFAE) mapping, FIRM and ECGI (External ECG vest) phase mapping, and CARTOFINDER have also been evolved to apply for the mapping strategies in humans recently. Each approach has its clinical advantages and inherits technical shortcomings [33]. Newer mapping systems have transformed the clinical electrophysiology field, and have enabled operators to overcome major limitations of the conventional mapping by using catheters with multiple-spine and multiple closely spaced small electrodes. The advanced mapping catheters facilitate the creation of more accurate maps through simultaneous acquisition of ten thousand points or maybe more. From the mechanistic perspective, the better map representation of both rapid organized sites (potential sources) and rapid fragmented sites (critical substrates) may clarify further the role and interplay between different arrhythmic mechanisms in the sustainment of AF, as well as the contribution of specific anatomical structures in supporting these mechanisms. In this study, two configurations of the advanced mapping catheters were applied to the models and the basket was not selected for the modeling, since the panoramic mapping using the basket catheter frequently showed uninterpretable mapping results [38]. Despite the existence of various technologies capable of visualizing AF drivers, the characterization of these drivers is quite different. In the FIRM mapping system, for example, drivers are displayed offline in a 2D system. Most of these drivers were found to be rotors and were observed to be stable and long-lasting with certain meandering of the core [39]. In the ECGI, the rotors are less stable, lasting at most 2–3 rotations but they demonstrate spatial consistency [40]. The system also identifies other focal source drivers. The observations of ECGI are actually quite consistent with driver mapping using both the CARTOFINDER [41, 42] and AcQMap modules [43]. Interestingly, electrographic flow mapping recently published [44] is as a new approach to detect action potential sources in atria of AF patients, which has a potential to distinguish between active and passive rotors.

The local efficiency plateau can be added on to future mapping and ablation strategy since it can serve as a metric for further identifying and classifying rotor specifically during the electrical remodeling. In Fig. 4d, an increase in the derivative of local efficiency as a function of information threshold indicates increased remodeling within a driver activation. The exact derivative value in Fig. 5 is distinct from prior simulation-based results, which indicated that exact remodeling thresholds should be validated based on clinical or animal model rotational activation data. However, the functional form of the local efficiency graph, given the lack of a distinct plateau in the 40–80% information threshold range, suggests a lack of underlining atrial remodeling. Therefore, despite the presence of rotational activation in the anterior wall region, such a rotor identification by the current AF mapping algorithm may be phenomenological. It might not be reliable for its mechanistic inference to classify it directly as AF driver, although the clinical analysis data is preliminary based on a single mapping data. More robust studies are necessary to determine how the local efficiency information can be successfully used in classifying the optimal ablation targets for AF termination.

Rotors with varying degrees of remodeling can therefore be identified based on the local efficiency derivative, with potential classifications corresponding to the degree of remodeling in the mapping region.

In future studies, targeting regions for ablation based in this identification can be explored, potentially providing an avenue for identifying remodeling-based ablation targets without requiring extensive MRI imaging prior to treatment [45]. Specifically, classification of ablation targets through the local efficiency metric should be validated based on pre-clinical and clinical outcomes by a supervised machine learning linear classifier. Simultaneous mapping in ex vivo human atria [46] with mapping and optical mapping might be pursued for a supervised machine learning algorithm to classify advanced mapping signals as driver or non-driver. Once correlation between local efficiency and successful AF termination has been established, the metric can be included into an integrated workflow to enhance the ability to determine the true mechanism of apparently focal activation. Combination of our modeling analysis through regional mutual information networks with high-density mapping systems and other algorithms may help to relate these peculiar signal features with specific underlying mechanisms (e.g., focal vs. reentrant activity, multiple wavelets).

### Limitations

One limitation of the approach as a standalone technique is the inability to precisely identify the source of AF driver: the distinction between rotational and irregular activation in a particular simulated region occurs at a range of catheter positions (Fig. 3). However, automated detection of rotors has previously been performed with advanced mapping catheters [42]. The proposed method could therefore be utilized in tandem with such existing techniques to enable identification of the degree of remodeling in already identified drivers, providing the electrophysiologist with additional information on the targets. Following remodeling identification areas of interest can be highlighted on global maps of AF wavefronts generated via stitching and ablated by creating a cluster of lesions immediately surrounding the target [42].

Further work with human data is required to translate these insights to clinical use. The activations analyzed in this report do not fully represent clinical AF dynamics, which have variable cycle lengths and can be obscured by remodeling mechanism not represented in this study, such as fibrotic substrate. Therefore, this study identifies a local efficiency pattern indicative of spontaneous Ca^2+^ release-based remodeling in rotational activation but does not explore how this signature may be obscured in clinical data. Furthermore, this simulation focuses on AP voltages, rather than unipolar or bipolar electrograms typically generated by commercial intracardiac mapping catheters. Both virtual AP and virtual electrogram features can be used to explore viable strategies for ablation [44, 47]. However, calculations of pseudo electrograms require assumptions about fiber diameters and the distance between the tissue and the electrode, which can be variable in practice [48]. APs were therefore utilized directly in this study to first establish if regional information networks displayed any distinguishing features due to driver activity. This study represents a necessary first step in identifying local efficiency as distinguishing feature of remodeled driver observable at the regional scale.

### Summary

The real-time mapping identification of electrical drivers within remodeled heart tissue is vital for the AF management. This study explored an information and graph theory approach to this identification, focusing on high-resolution regional cardiac mapping. We demonstrate in two computational models that a decrease in the derivative of local efficiency as a function of information threshold is characteristic of driver in high resolution maps (2 mm electrode spacing). This metric is a measure of regional fault tolerance in information transfer and therefore provides insight into myocardial communication on the local scale. Further simulations with tissues at various degrees of remodeling suggest that the local efficiency derivative correlates with rotational activity within remodeled tissue. The study suggests an approach to atrial remodeling driver identification, therefore a possible focal substrate mapping and ablation strategy.

## Supplementary Information

Below is the link to the electronic supplementary material.

Video S1: The binary video based on the Karma model utilized in the 2D analysis obtained following spiral wave break up (key simulation parameters: nb =1.0 , m = 7.0, eps = 0.4). (AVI 53457 KB)

Video S2: The binary video based on the Karma model utilized in the 2D analysis obtained in the spiral wave regime (key simulation parameters: nb =1.0 , m = 7.0, eps = 0.4). (AVI 20897 KB)

Video S3: The binary video based on a 3D AF-remodeled tissue model utilized in the analysis of chaotic activation (threshold at -50mV). (AVI 2919 KB)

Video S4: The binary video based on a 3D AF-remodeled tissue model utilized in the analysis of rotational activation (threshold at -50mV). (AVI 2351 KB)

Video S5: Clinical recording (30 seconds) of rotational activation from the subject 841-004 (a pesistent AF patient) by Shakozy et al 202025 A rotational activation in the left atrium anterior wall region was identified by the CARTOFINDER algorithm in the CARTO 3 mapping system & diagnosed by the physician as a rotor. (AVI 87540 KB)

Video S6: Clinical recording (~ 20 seconds) binary video fragment utilized in analysis The binary version of a segment of video S5 converted using the -153ms LAT threshold. (AVI 10919 KB)

Supplementary file7 (PDF 486 KB)
