# Supplementary material for: Atrial fibrillation driver identification through regional mutual information networks: a modeling perspective
Source: J Interv Card Electrophysiol. 2022 Jan 4;64(3):649–60. doi: 10.1007/s10840-021-01101-z (PMC9470649; doi:10.1007/s10840-021-01101-z)
Supplement: Supplementary file 7 — Supplementary file7 (PDF 486 KB) [file 10840_2021_1101_MOESM7_ESM.pdf]

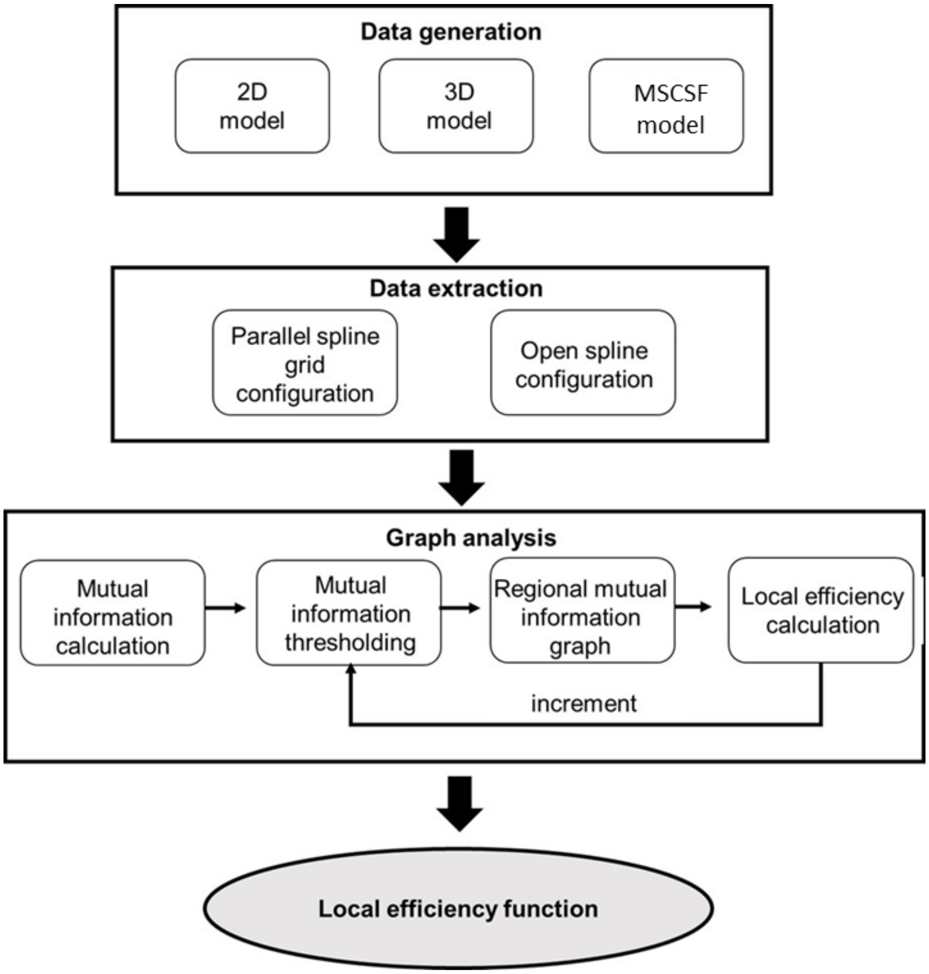

Figure S1: Schematic illustrating the processes of arriving at a local efficiency as a function of mutual information threshold for varied models and catheter spline configurations.

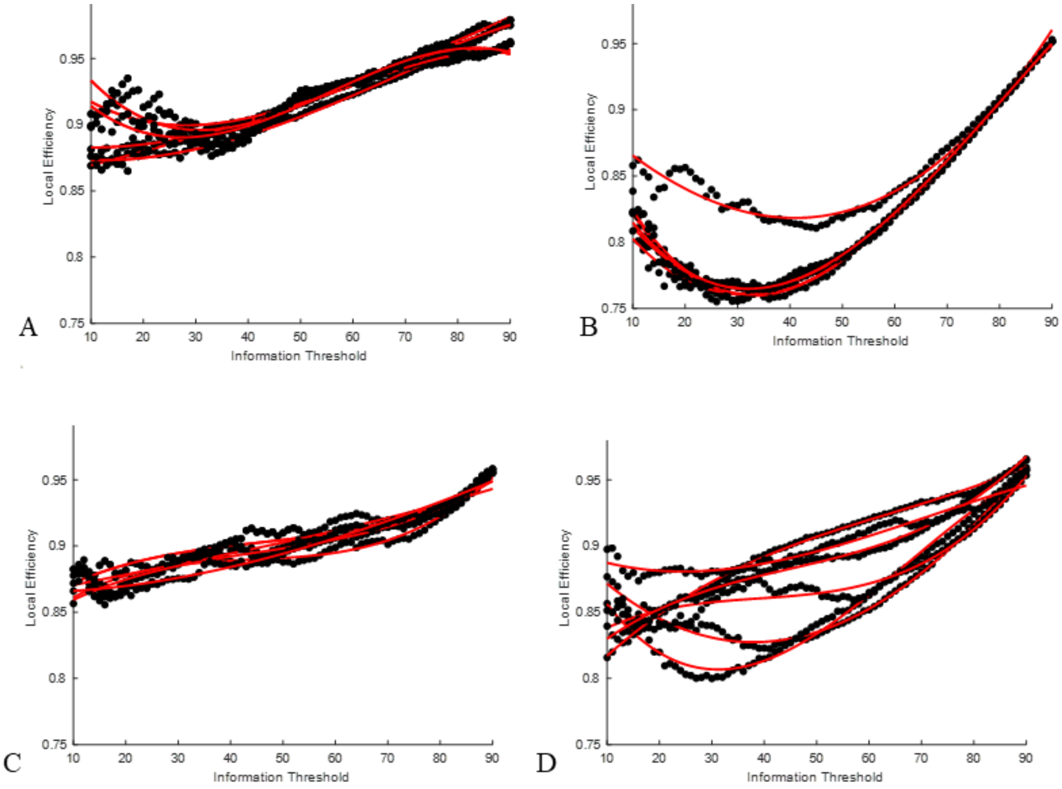

Figure S2: Local efficiency functions and polynomial fits. A) Local efficiency and third-degree polynomial fit for six parallel spline catheter positions in the 3D irregular simulation. B) Local efficiency and third-degree polynomial fit for six parallel spline catheter positions in the 2D irregular simulation. C) Local efficiency and third-degree polynomial fit for six parallel spline catheter positions in the 3D rotational simulation. D) Local efficiency and third-degree polynomial fit for six parallel spline catheter positions in the 2D rotational simulation.

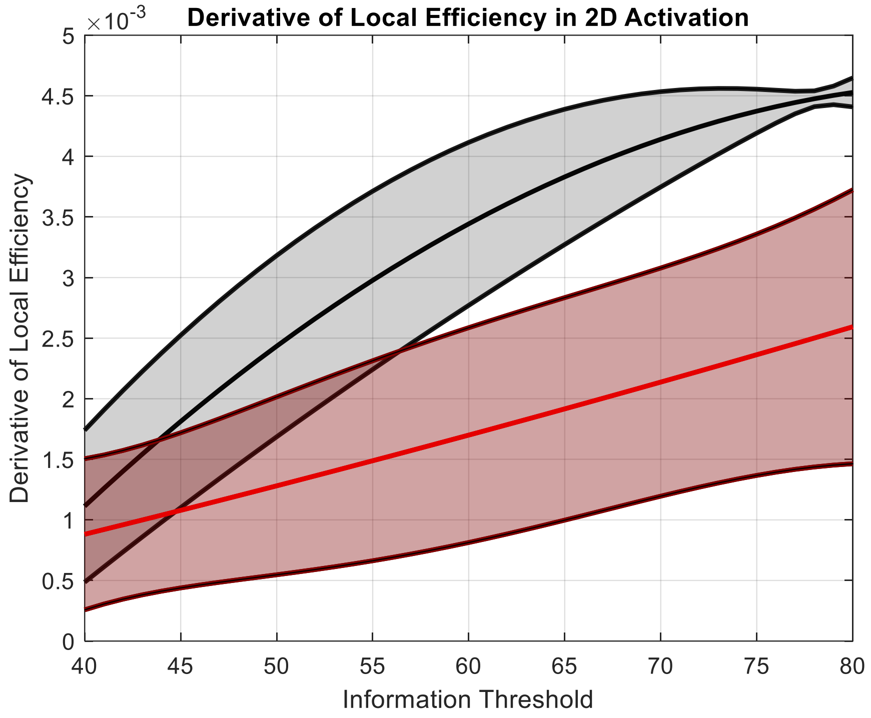

Figure S3: Local efficiency derivative comparison for the 2D Karma simulation. The irregular activation simulation results are shown in black and the rotational simulation results are shown in red. Shaded region represents the 95% confidence interval.

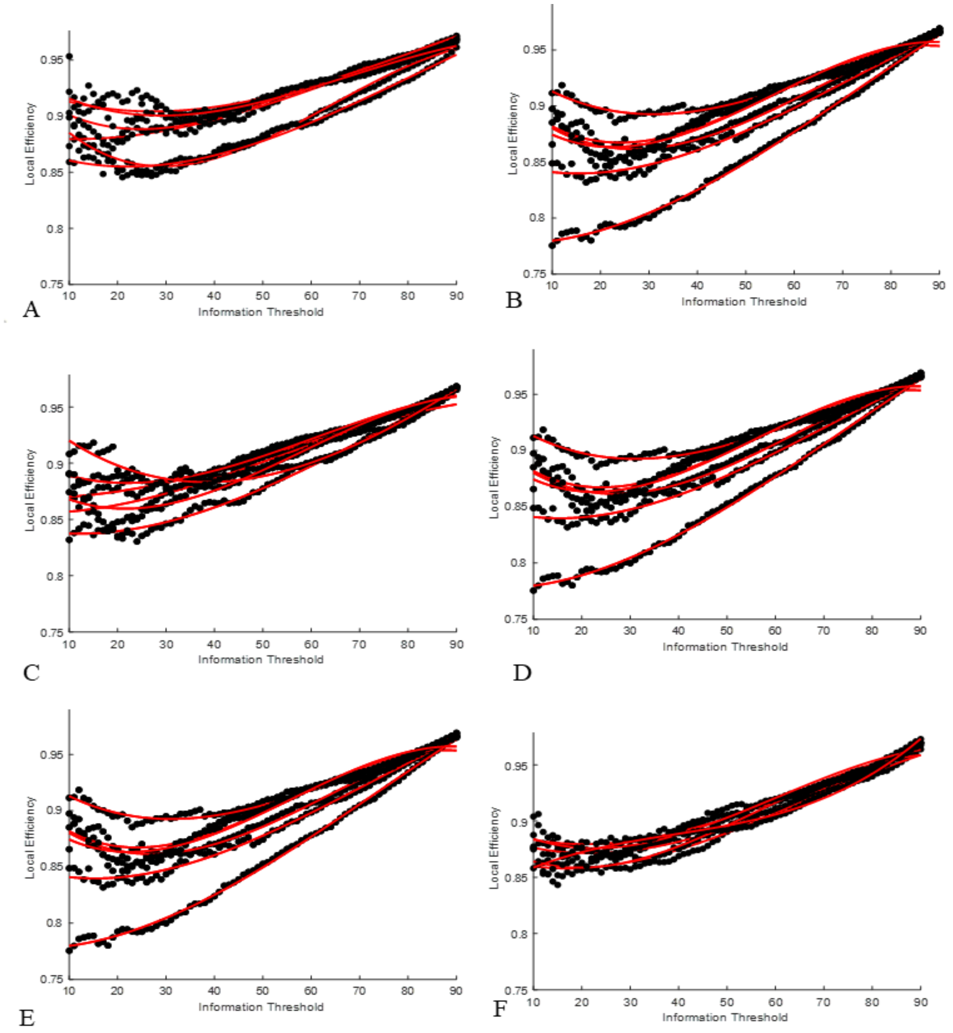

Figure S4: Local efficiency functions and polynomial fits for simulations with varied degrees of remodeling. A) Local efficiency and third-degree polynomial fit for six parallel spline catheter positions in a 10% remodeling simulation. B) Local efficiency and third-degree polynomial fit for six open spline catheter positions in a 10% remodeling simulation. C) Local efficiency and third-degree polynomial fit for six parallel spline catheter positions in a 50% remodeling simulation. D) Local efficiency and third-degree polynomial fit for six open spline catheter positions in a 50% remodeling simulation. E) Local efficiency and third-degree polynomial fit for six parallel spline catheter positions in a 90% remodeling simulation. F) Local efficiency and third-degree polynomial fit for six open spline catheter positions in a 90% remodeling simulation.
